# Mindful Parenting Mediated Between Mothers’ Perceived Stress During COVID-19 and Child Adjustment

**DOI:** 10.1007/s12671-022-02018-y

**Published:** 2022-11-07

**Authors:** Rebecca Y. M. Cheung, Iris Yili Wang

**Affiliations:** 1 School of Psychology and Clinical Language Sciences, University of Reading, Reading, UK; 2 Department of Early Childhood Education, The Education University of Hong Kong, Hong Kong, China

**Keywords:** Mothers’ stress during COVID-19, Mindful parenting, Child adjustment

## Abstract

**Objectives:**

Maternal stress is associated with a myriad of maladjusted outcomes among children. To identify the role of mindful parenting between mothers’ stress and child adjustment during the COVID-19 pandemic, this study tested competing hypotheses with mothers’ mindful parenting as a mediator versus a moderator.

**Methods:**

A total of 172 Chinese mothers of preschool-aged children participated in this study. Participants completed a self-report measure of stress during COVID-19 and mindful parenting, as well as a mother-report measure of children’s prosocial behavior, internalizing problems, and externalizing problems. Structural equation models were conducted to examine the mediation versus moderation effects of mindful parenting between mothers’ stress during COVID-19 and child adjustment, after controlling for family income, children’s age, sex, and adjustment at baseline.

**Results:**

Findings indicated that mindful parenting mediated the link between mothers’ stress during COVID-19 and child adjustment, including internalizing problems, externalizing problems, and prosocial behavior. A test of competing hypothesis showed that mindful parenting did not moderate between mothers’ stress during COVID-19 and child adjustment.

**Conclusions:**

This study revealed the mediating effects of mindful parenting between mothers’ perceived stress during COVID-19 and child adjustment. The findings inform researchers and practitioners about mindful parenting as a potential mechanism between maternal stress and child adjustment during the pandemic.

The global coronavirus disease 2019 (COVID-19) pandemic has a devastating effect on families given its health, financial, and social implications (Brown et al., 2020; Chung et al., 2020; Zafar et al., 2021). Although public health measures imposed by governments worldwide have reduced the spread of the virus, family challenges have continued to emerge. In China, for instance, the government has implemented policies such as physical distancing (e.g., closure of schools and community centers), isolation of patients and their close contacts, border controls (e.g., flight circuit, post-entry quarantine), regular COVID-19 mass testing, and digital contact tracing (Civil Aviation Administration of China, 2020; Chinese Center for Disease Control & Prevention Weekly, 2020, 2021). While these policies have led to smaller outbreaks between mid-2020 and the end of 2021 (National Health Commission of China, 2020; 2021; Zhou et al., 2021), they have brought challenges to families, including disrupted family routines, reduced family support such as babysitters, reduced work-life balance, and unemployment (Chen et al., 2022; Guo et al., 2021; Zhao et al., 2020). School closure and home confinement have also reduced children’s interactions with teachers and peers (G. Wang et al., 2020). During the pandemic, parents may be especially stressful, as they have to take on additional responsibilities to meet the health, educational, and social demands of children (Adams et al., 2021).

According to the Risky Families Model (Repetti et al., 2002, 2012), stressful environments may compromise child adjustment through a cascade of processes, including stressful family interactions and negative parenting practices. The stressful environment in which families dwell necessitates parents’ constant efforts and responsive coping abilities (Repetti et al., 2002). Such an ongoing and heavy burden may alter parenting behavior towards a negative direction, which, in turn, brings on children’s internalizing and externalizing problems (Repetti et al., 2002, 2012). For instance, a longitudinal study involving 2606 families showed that mothers’ perceived stress undermined their warmth during mother–child interactions, which was further linked to children’s externalizing problems (Flannery et al., 2021). In connection to COVID-19, Prime et al. (2020) further presented a conceptual framework to address how the pandemic may affect child development via cascading effects of social disruption, caregivers’ stress, and family well-being (see also Feinberg et al., 2021). In the face of elevated pandemic-related stress, such as financial strain, disruption in daily routines, and children’s challenging social and academic demands (Brown et al., 2020), parents may exhibit greater negative parenting behavior, such as reacting emotionally towards children’s misbehavior (Prime et al., 2020). Indeed, recent studies have demonstrated that parents’ perceived stress during COVID-19 was associated with poorer parenting behavior, such as coercive and harsh parenting (e.g., scolding, spanking, and yelling at children; Chung et al., 2020; Giannotti et al., 2022; Lucassen et al., 2021). Parents’ perceived stress during COVID-19 was also positively linked to children’s emotional and behavioral problems (Cohodes et al., 2021; Giannotti et al., 2022; Spinelli et al., 2020, 2021; Sun et al., 2022). Moving beyond the direct association between parental stress and child adjustment during COVID-19, studies have further identified mediating mechanisms involving parenting behavior, such as parental involvement and autonomy support (C. Wang et al., 2022). Grounded in theoretical and empirical research of family risks (e.g., Prime et al., 2020; Repetti et al., 2002), parenting may thus serve as a process between parental stress and child adjustment during the pandemic.

Mindful parenting may be a potential mediating mechanism between parents’ stress during COVID-19 and child adjustment. Mindful parenting refers to the integration of mindfulness into parenting behavior (Duncan et al., 2009). Through a mindful approach, parents are more compassionate, nonjudgmental, and self-regulating in parenting (Duncan et al., 2009). They are also more likely to develop emotional awareness and listen with full attention to themselves and their children (Duncan et al., 2009). Although a handful of studies have shown that parenting stress was associated with a lower level of mindful parenting (Cheung et al., 2019; Fernandes et al., 2021; Moreira et al., 2019), little has been done to examine the role of stress in mindful parenting during the pandemic. Based on theories of family risks and social disruption (e.g., Prime et al., 2020; Repetti et al., 2002), the stressful everyday childrearing environment may undermine parents’ health and positive parenting behavior (Flannery et al., 2021). While acute fight or flight responses may be evolutionarily adaptive (e.g., to attack or escape from an alarming virus), chronic fight or flight reactions may be maladaptive for parents who feel particularly threatened. For instance, longstanding research has indicated that prolonged activation of stress hormones disrupts the modulation of response systems, thereby worsening people’s physical health, mental health, and parenting quality (Adam et al., 2017; Bos et al., 2018; Franz et al., 2021). In the face of COVID-19, parents may be preoccupied with self-directed and family-directed concerns, such as shortage of groceries, cleaning supplies, and face masks; disruption of family routines; and personal safety (Prentice et al., 2022; Taylor et al., 2020). As core stressors, these concerns may, in turn, reduce parents’ capabilities to be mindful in parenting, e.g., to listen to themselves and their child with full attention, to regulate their own behavior, and to be emotionally aware of the needs of themselves and their child. A lower level of mindful parenting may be further associated with children’s poorer adjustment outcomes (Bögels & Restifo, 2013; Potharst et al., 2021). In contrast, when parents are mindful, they are more capable of pausing, disengaging from automatic reactions to children’s misbehavior, and reducing judgments during parent–child interactions (Bögels & Restifo, 2013). They are also more likely to respond skillfully (versus react automatically) to their needs and the needs of their child (Bögels & Restifo, 2013; Duncan et al., 2009). With poise and compassion, parents who are more mindful in parenting may be more likely to set an example for their children to practice self-regulation (Cheung et al., 2021; Sameroff, 2010), thereby promoting child adjustment. Based on the literature, mindful parenting may serve as a mediating mechanism between parental stress and child adjustment.

Drawing from models of risk and resilience (Bonanno et al., 2010; Masten, 2001; Masten & Narayan, 2012), mindful parenting may also serve as a moderator to ameliorate the negative effect of parental stress on child adjustment. In their conceptual framework of social disruption and child adjustment, Prime et al. (2020) posited that some families may be more vulnerable to the influence of the pandemic. For instance, pre-existing family vulnerabilities such as mental health challenges and poverty may exacerbate the negative sequelae of the pandemic. On the contrary, pre-existing positive family functioning such as positive parent–child relationships may buffer or ameliorate the negative effects of the pandemic on child adjustment (Masten & Narayan, 2012; Prime et al., 2020). Supporting the theoretical models, a recent study indicated that parents’ greater practice of emotional coaching (e.g., helping children be aware of, express, and deal with negative emotions) weakened the association between parental stress during COVID-19 and child maladjustment (Cohodes et al., 2021). Zooming in on the practice of mindful parenting, mothers’ greater mindful parenting attenuated the link between socioeconomic adversity and children’s negative development outcomes, such as sleep/wake problems (Kelly et al., 2022). Indeed, mindfulness allows parents to be aware of their challenges nonjudgmentally and regulate their automatic parenting behavior (Duncan et al., 2009; Parent et al., 2016; Pothrast et al., 2019; 2021; Y. Ren et al., 2021). By disengaging themselves from autopilot, parents are supported to be attentive to parent–child experiences arising in the present moment, amid stressful circumstances such as the COVID-19 pandemic or other adversities. As such, mindful parenting may attenuate the potentially negative effect of parental stress during COVID-19 on child adjustment.

Despite the importance of both mothers and fathers in child development (Li & Lamb, 2015), mothers play a critical role in childrearing in the Chinese context (Dou et al, 2020; Tam, 2009). According to a time use study involving 2008 families from multiple provinces in China, mothers of children under 6 years old typically spent 3.05 hours a day in childcare, whereas fathers typically spent 0.92 hours a day in childcare (F. Du et al., 2018). That is, mothers spent more time in providing childcare than did fathers among Chinese families (F. Du et al., 2018). Meanwhile, the employment rates of mothers and fathers were 69% and 86%, respectively (F. Du et al., 2018). As such, although a majority of men and women are employed in the workforce, mothers remain to be the primary caregivers in China (see also National Bureau of Statistics of China, 2019). Recent studies have demonstrated the significance of mothers’ behavior in child adjustment in the Chinese context. For instance, compared to fathers, mothers’ emotion dysregulation had a stronger effect on their partners’ and their children’s emotion dysregulation (Cheung et al., 2020). Similarly, the relation between negative parenting practices (e.g., physical control) and children’s externalizing behavioral problems was stronger for mothers than for fathers (Han et al., 2021). Moreover, mothers’, but not fathers’, exercise of psychological control, such as guilt induction and love withdrawal, was negatively associated with adult children’s social-emotional development (Xing et al., 2017). Given that mothers have remained to be the primary caregivers in Chinese families (F. Du et al., 2018), it is crucial to investigate how the pandemic has affected their levels of stress, parenting behavior, and children’s adjustment outcomes.

The present study aims to examine competing hypotheses of mediation versus moderation effects of mindful parenting between mothers’ stress during COVID-19 and child adjustment in the Chinese context, including internalizing problems, externalizing problems, and prosocial behavior, over and above covariates including children’s age and sex, household income, as well as children’s baseline adjustment. Building on frameworks of family risks and recent findings (e.g., Cohodes et al., 2021; Giannotti et al., 2022; Prime et al., 2020; Repetti et al., 2002), we hypothesized that mindful parenting would mediate the link between mothers’ stress during COVID-19 and child adjustment (see Fig. 1). Alternatively, drawing from risks and resilience framework (e.g., Masten, 2001; Masten & Narayan, 2012; Prime et al., 2020), we hypothesized that mindful parenting would moderate the link between mothers’ stress during COVID-19 and child adjustment (see Fig. 2).

**Fig. 1:**
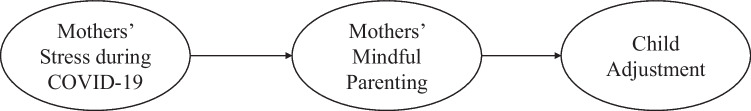
Conceptual model of mindful parenting as a mediator between mothers’ stress during COVID-19 and child adjustment

**Fig. 2:**
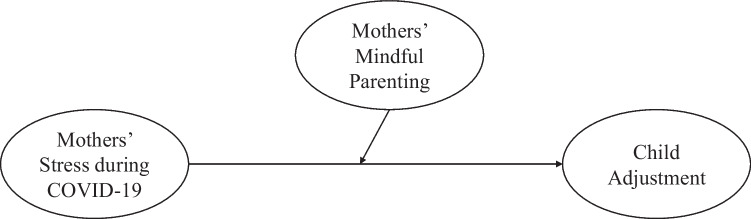
Conceptual model of mindful parenting as a moderator between mothers’ stress during COVID-19 and child adjustment

## Method

### Participants

A total of 172 Chinese mothers (*M*_age_ = 33.82 years, *SD* = 3.54 years) of preschool-aged children (51.05% girls, *M*_age_ = 6.80 years, *SD* = 2.33 years) were recruited online. Baseline assessment of child adjustment was collected between August 2020 and November 2020. Follow-up data on mothers’ stress during COVID-19, mindful parenting, and child adjustment were collected 6 months later from February 2021 to May 2021. The median monthly household income was RMB¥20,000.00 (*SD* = RMB¥33,101.57), i.e., ~ US$2,933.80 (*SD* = US$5,223.87). Participants were from 14 provinces (Fujian, Guangdong, Heilongjiang, Hubei, Jiangsu, Jiangxi, Liaoning, Shanxi, Zhejiang), one autonomous region (Nei Mongol), three direct-administered municipalities (Beijing, Shanghai, and Tianjin), and one special administrative region of China (Hong Kong). Although the median monthly household income of the present sample was higher than the average monthly income of the general urban population (National Bureau of Statistics of China, 2021), it is lower than the median monthly household income of Hong Kong (Census & Statistics Department, 2021). Further investigations showed that the median monthly household income of the present sample was similar to that of the previous studies involving urban citizens of Mainland China and Hong Kong (e.g., Cheung & Chung, 2022; L. Ren et al., 2020; X. Wang et al., 2021; Yan et al., 2021). A total of 71.83% of the mothers were employed full-time, 7.04% were employed part-time, and 21.13% were unemployed. In terms of education, 2.80% of the mothers reported that they completed junior high school, 2.80% completed high school, 5.59% had a diploma or associate degree, 74.83% had a bachelor’s degree, and 13.98% had a graduate degree. All participants were biological mothers and three mothers reported that they were divorced. The average household size of the current sample was 4.24 (*SD* = 1.26).

### Procedures

Participants were recruited online via online platforms and forums. At baseline, the participating mothers provided their contact information, such that the trained research assistants could contact them for the follow-up assessment. Upon informed consent, participants were directed to an online questionnaire, which took approximately 20 minutes to complete. The data collected from the participants were held in the strictest confidentiality. No incentives were offered to participants who responded to the study.

### Measures

#### Mothers’ Stress During COVID-19

Mothers’ stress during COVID-19 was assessed by an adapted 6-item measure developed by Brown et al. (2020). Mothers responded to whether they had experienced the following stressors as a result of social restrictions, childcare closures, and school childcare closures during the outbreak of COVID-19: (a) parent mood/stress, (b) parent physical health, (c) parent’s relationship/interactions with partner, (d) parent’s relationship/interactions with child(ren), (e) child(ren’s) physical health, and (f) child(ren’s) academic/learning on a scale from 1 (*never*) to 5 (*almost always*). The raw scores were averaged, with higher raw scores of each item indicating mothers’ greater stress during COVID-19. The measure was translated from English to Chinese by trained research assistants following the back-translation procedures (Brislin, 1970). Cronbach’s *α* and McDonald’s *ω* of this measure were 0.87 and 0.89, respectively.

#### Mindful Parenting

The 29-item Interpersonal Mindfulness in Parenting (IMP) questionnaire (de Bruin et al., 2014; Duncan et al., 2009) was used to assess mothers’ mindful parenting behavior on a scale from 1 (*never true*) to 5 (*always true*). The IMP had 6 subscales, namely (a) listening with full intention, (b) nonjudgmental acceptance of parental functioning, (c) emotional awareness of child, (d) compassion for child, (e) emotional awareness of self, and (f) emotional non-reactivity in parenting. The scale has been previously translated to Chinese and validated in a sample of Chinese parents (Lo et al., 2018). Sample items included, “Pausing before reacting in difficult situations with the child” and “Paying close attention to the child when spending time together.” The raw scores of 14 of the 29 items reversed, as they reflected the opposite of mindful parenting. The scores of the items were then averaged to form subscale scores, with greater scores indicating greater mindful parenting behavior. Cronbach’s *α* and McDonald’s *ω* were 0.73 and 0.75 for listening with full intention (5 items), 0.65 and 0.69 for nonjudgmental acceptance of parental functioning (6 items), 0.57 and 0.60 for emotional awareness of child (3 items), 0.84 and 0.84 for compassion for child (6 items), 0.71 and 0.71 for emotional awareness of self (4 items), and 0.80 and 0.81 for emotional non-reactivity in parenting (5 items), respectively.

#### Children’s Internalizing and Externalizing Problems, and Prosocial Behavior

The 25-item Strengths and Difficulties Questionnaire (SDQ; Goodman, 1997) was used to measure mothers’ report of children’s prosocial behavior, externalizing problems, and internalizing problems on a 3-point scale from 1 (*not true*) to 3 (*certainly true*). The measure had been translated into Chinese and validated in samples of parents from Mainland China and Hong Kong (Y. Du et al., 2008; Lai et al., 2010). Sample items included, “[my child is] considerate of other people’s feelings” (prosocial behavior), “[my child] often loses temper” (externalizing problems), and “[my child is] often unhappy, depressed or tearful” (internalizing problems). After reversing the raw scores of negatively worded items, the scores of each subscale were averaged, with higher scores indicating greater prosocial behavior, externalizing problems, and internalizing problems, respectively. Cronbach’s *α* and McDonald’s *ω* were 0.72 and 0.72 for prosocial behavior, 0.74 and 0.77 for externalizing problems, and 0.64 and 0.65 for internalizing problems, respectively. At baseline, Cronbach’s *α* and McDonald’s *ω* were 0.72 and 0.70 for prosocial behavior, 0.74 and 0.70 for externalizing problems, and 0.70 and 0.78 for internalizing problems.

Given that children’s physical interactions with peers were restricted due to school closure and social distancing (e.g., Fegert et al., 2020; The Government of Hong Kong special Administrative Region Press Releases, 2020), two items of prosocial behavior subscale, namely “[my child] shares readily with other children, for example toys, treats, pencils” and “[my child is] kind to younger children,” were removed in the supplementary analyses. The retained items included “[my child is] considerate of other people’s feelings,” “[my child is] helpful if someone is hurt, upset or feeling ill,” and “[my child] often offers to help others (parents, teachers, other children).” The three items were included, as they reflected prosocial behavior towards other people, regardless of their age and the context. The prosocial behavior measure with the removed items had Cronbach’s *α* and McDonald’s *ω* = 0.66 and 0.67 at baseline, respectively, and 0.59 and 0.59 at the follow-up, respectively.

### Data Analyses

Correlations, means, and standard deviations of the manifest variables in the structural equation models were computed. Structural equation modeling was then conducted using MPLUS, Version 8.7 (Muthén & Muthén, 1998–2017) to investigate the mediating versus moderating effects of mindful parenting between mothers’ stress during COVID-19 and child adjustment, with household income, children’s sex, and children’s age as covariates of child adjustment.

For the mediation model, a post hoc power analysis using semPOWER (Moshagen & Erdfelder, 2016) was conducted to detect the power with *N* = 172, *df* = 163, RMSEA = 0.05, and alpha = 0.05. The findings indicated a power of 94.95% to reject the null hypothesis (i.e., the “wrong” model) with the degree of misspecification corresponded with RMSEA = 0.05 on alpha = 0.05. Given the bootstrapping method yields more accurate estimates of the indirect effect standard errors compared to other approaches (Shrout & Bolger, 2002), it was used to determine the mediation effects. In addition to testing mindful parenting as a mediator, additional analyses were conducted to test the alternative directionality of effects, with mothers’ stress during COVID-19 as a mediator, given previous research only indicated the cross-sectional relations between parental stress during COVID-19 and parenting behavior (e.g., Chung et al., 2020; Giannotti et al., 2022).

As for the moderation model, when *N* = 172, *df* = 277, RMSEA = 0.05, and alpha = 0.05, the power was 99.53% to reject the null hypothesis, with the degree of misspecification corresponded with RMSEA = 0.05 on alpha = 0.05. With reference to previous research (e.g., Cheung et al., 2018; Merrilees et al., 2011), the interaction terms were manually created by multiplying the values between the subscales of each latent construct, e.g., “Listening with Full Attention” (i.e., subscale of mindful parenting) × “Parent Mood / Stress” (i.e., subscale of mothers’ stress during COVID-19); “Nonjudgmental Acceptance” (i.e., subscale of mindful parenting) × “Parent Physical Health” (i.e., subscale of mothers’ stress during COVID-19). To verify the findings, a second model was conducted by adding a latent interaction term between mothers’ stress during COVID-19 and mindful parenting within the MPLUS environment.

For both models of mediation and moderation, analyses were conducted separately using the original measure of prosocial behavior and its shortened version, given that the items on peer interactions might have been less relevant due to school closure and social distancing.

## Results

Table 1 shows the means, standard deviations, and correlations among the variables under study.

**Table 1:**
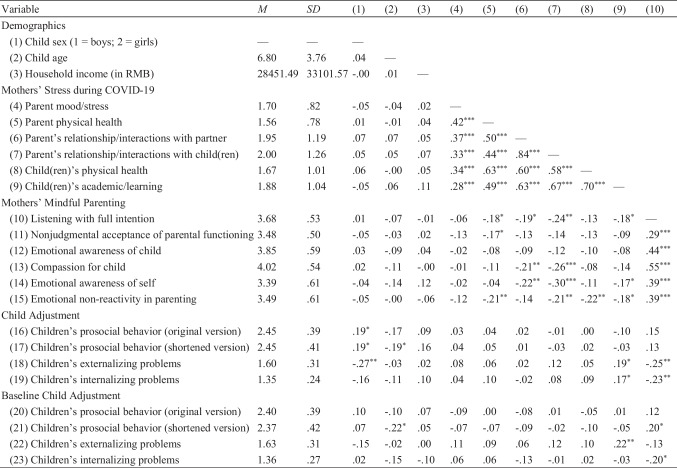

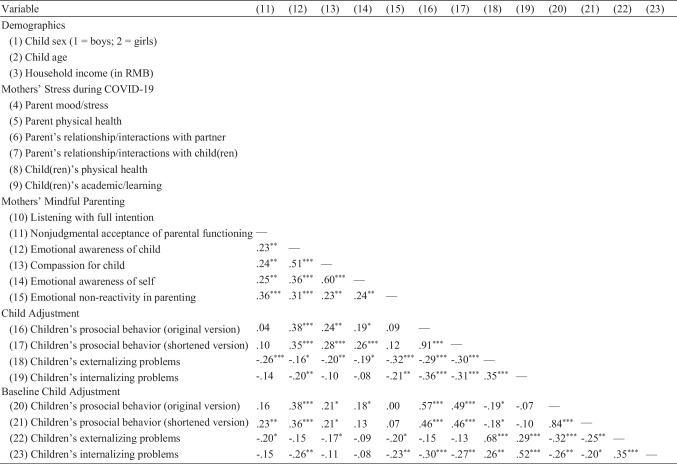
Means, standard deviations, and correlations of the variables

^*^p < .05, ^**^p < .01, ^***^p < .001. Items ranged from 1 (*never*) to 5 (*almost always*) for maternal stress during COVID-19, from 1 (*never true*) to 5 (*always true*) for mindful parenting, and from 1 (*not true*) to 3 (*certainly true*) for child adjustment

### Mindful Parenting as a Mediator

**Fig. 3:**
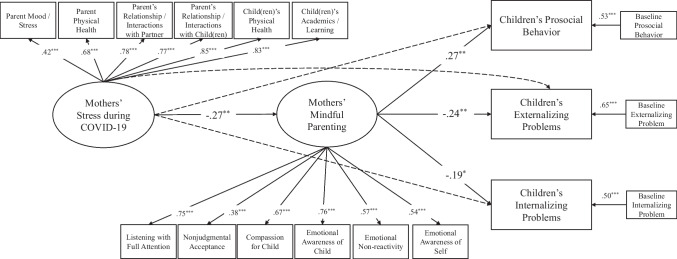
Final model of mindful parenting as a mediator between mothers’ stress during COVID-19 and child adjustment outcomes. This model reflects the results based on the original measure of children’s prosocial behavior. *χ*^2^(163) = 230.73, *p* < 0.001, CFI = 0.93, TLI = 0.92, RMSEA = 0.05. Household income, children’s age, and children’s sex were included as covariates but are not depicted in the figure for clarity. Non-significant paths are depicted in the dashed arrows. *p < 0.05, **p < 0.01, ***p < 0.001

The structural equation model fit adequately to the data (*χ*^2^(163) = 230.73, *p* < 0.001, CFI = 0.93, TLI = 0.92, RMSEA = 0.05). In the measurement model, the latent variables of mothers’ stress during COVID-19 and mindful parenting were significantly associated with the manifest variables involving the respective subscales (*p*s < 0.001), respectively. As for the structural model, mothers’ stress during COVID-19 was negatively related to mindful parenting (*β* =  − 0.27, *p* < 0.01). Mothers’ mindful parenting, in turn, was related to child adjustment, including greater prosocial behavior (*β* = 0.27, *p* < 0.01), fewer externalizing problems (*β* =  − 0.24, *p* < 0.01), and fewer internalizing problems (*β* =  − 0.19, *p* < 0.05), after controlling for children’s baseline prosocial behavior, externalizing problems, and internalizing problems (*p*s < 0.001). Children’s sex, children’s age, and household income were entered as covariates of the variables under study (see Fig. 3 and Table 2 for details). Based on 10,000 bootstrap samples with replacement, the 95% confidence interval (CI) indicated that the standardized indirect effects between mothers’ stress during COVID-19 and children’s prosocial behavior, externalizing problems, and internalizing problems did not include zeros (CI_prosocial behavior_: (− 0.17, − 0.01); CI_externalizing problems_: (0.02, 0.15); CI_internalizing problems_: (0.01, 0.15)). Therefore, mindful parenting mediated between mothers’ stress during COVID-19 and child adjustment, including prosocial behavior, externalizing problems, and internalizing problems.

**Table 2:** Parameter estimates of the mediation model

Parameter	Unstandardized *B* (*SE*)	Standardized *β*
*Measurement model*		
Mothers’ stress during COVID-19		
→ Parent mood/stress	1.00^f^	.75^***^
→ Parent physical health	.47 (.12)	.38^***^
→ Parent’s relationship/interactions with partner	.99 (.14)	.67^***^
→ Parent’s relationship/interactions with child(ren)	1.05 (.14)	.76^***^
→ Child(ren)’s physical health	.86 (.15)	.57^***^
→ Child(ren)’s academic/learning	.82 (.14)	.54^***^
Mothers’ mindful parenting		
→ Listening with full intention	1.00^f^	.42^***^
→ Nonjudgmental acceptance of parental functioning	1.51 (.33)	.68^***^
→ Emotional awareness of child	2.69 (.56)	.78^***^
→ Compassion for child	2.85 (.60)	.77^***^
→ Emotional awareness of self	2.61 (.53)	.85^***^
→ Emotional non-reactivity in parenting	2.51 (.52)	.83^***^
*Structural model*		
Maternal stress during COVID-19		
→ Mothers’ mindful parenting	− .31 (.13)	− .27^**^
→ Children’s prosocial behavior	.16 (.09)	.14
→ Children’s externalizing problems	− .02 (.06)	− .02
→ Children’s internalizing problems	.04 (.05)	.05
Mothers’ mindful parenting		
→ Children’s prosocial behavior	.25 (.08)	.27^**^
→ Children’s externalizing problems	− .18 (.06)	− .24^**^
→ Children’s internalizing problems	− .11 (.05)	− .19^*^
Autoregressive control variables		
Children’s baseline prosocial behavior		
→ Children’s prosocial behavior	.52 (.07)	.54^***^
Children’s baseline externalizing problems		
→ Children’s externalizing problems	.64 (.06)	.65^***^
Children’s baseline internalizing problems		
→ Children’s internalizing problems	.40 (.06)	.49^***^
Control variables		
Child’s sex (1 = boys; 2 = girls)		
→ Mothers’ mindful parenting	.00 (.07)	.01
→ Children’s prosocial behavior	.11 (.05)	.14^*^
→ Children’s externalizing problems	− .11 (.04)	− .18^*^
→ Children’s internalizing problems	− .08 (.03)	− .17^*^
Child’s age		
→ Mothers’ mindful parenting	− .01 (.01)	− .10
→ Children’s prosocial behavior	− .01 (.01)	− .10
→ Children’s externalizing problems	− .00 (.01)	− .04
→ Children’s internalizing problems	− .01 (.00)	− .10
Household income		
→ Mothers’ mindful parenting	.02 (.03)	.04
→ Children’s prosocial behavior	− .00 (.02)	− .01
→ Children’s externalizing problems	.02 (.02)	.06
→ Children’s internalizing problems	.05 (.02)	.22^**^
Covariance		
Mothers’ stress during COVID-19		
←→ Children’s sex (1 = boys; 2 = girls)	.00 (.02)	.01
←→ Children’s age	.03 (.12)	.02
←→ Family income	− .02 (.03)	− .06

^*^p < 0.05, ^**^p < 0.01, ^***^p < 0.001. The results presented in Table 2 are based on the original measure of children’s prosocial behavior

In the supplementary analyses, the structural equation model with excluded items of prosocial behavior fit adequately to the data (*χ*^2^(163) = 221.43, *p* = 0.002, CFI = 0.94, TLI = 0.93, RMSEA = 0.05). In the measurement model, the latent variables of mothers’ stress during COVID-19 and mindful parenting were significantly associated with the manifest variables involving the respective subscales (*p*s < 0.001), respectively. As for the structural model, mothers’ stress during COVID-19 was negatively related to mindful parenting (*β* =  − 0.27, *p* < 0.01). Mothers’ mindful parenting, in turn, was related to child adjustment, including greater prosocial behavior (*β* = 0.31, *p* < 0.001), fewer externalizing problems (*β* =  − 0.24, *p* = 0.001), and fewer internalizing problems (*β* =  − 0.19, *p* < 0.05), after controlling for children’s baseline prosocial behavior, externalizing problems, and internalizing problems (*p*s < 0.001). In addition, mothers’ stress during COVID-19 was positively related to children’s prosocial behavior (*β* = 0.17, *p* < 0.05). Children’s sex, children’s age, and household income were entered as covariates of the variables under study. Based on 10,000 bootstrap samples with replacement, the 95% CI indicated that the standardized indirect effects between mothers’ stress during COVID-19 and children’s prosocial behavior, externalizing problems, and internalizing problems did not include zeros (CI_prosocial behavior_: (− 0.20, − 0.02); CI_externalizing problems_: (0.02, 0.15); CI_internalizing problems_: (0.01, 0.14)). Therefore, mindful parenting mediated between mothers’ stress during COVID-19 and child adjustment, including prosocial behavior, externalizing problems, and internalizing problems.

### Test of Alternative Directionality of Effects: Mothers’ Stress During COVID-19 as a Mediator

The structural equation model fit adequately to the data (*χ*^2^(163) = 230.73, *p* < 0.001, CFI = 0.93, TLI = 0.92, RMSEA = 0.05). In the measurement model, the latent variables of mothers’ stress during COVID-19 and mindful parenting were significantly associated with the manifest variables involving the respective subscales (*p*s < 0.001), respectively. As for the structural model, the exogenous variable of mindful parenting was negatively related to mothers’ stress during COVID-19 (*β* =  − 0.27, *p* < 0.01). Mothers’ stress during COVID-19, however, was not related to child adjustment, including prosocial behavior (*β* = 0.14, *p* = 0.06), externalizing problems (*β* =  − 0.02, *p* = 0.80), and internalizing problems (*β* = 0.05, *p* = 0.48), after controlling for children’s baseline prosocial behavior, externalizing problems, and internalizing problems (*p*s < 0.001). Children’s sex, children’s age, and household income were entered as covariates of the variables under study. Hence, mothers’ stress during COVID-19 did not mediate between mindful parenting and child adjustment.

In the supplementary analyses, the structural equation model with excluded items of prosocial behavior fit adequately to the data (*χ*^2^(163) = 221.43, *p* < 0.001, CFI = 0.94, TLI = 0.93, RMSEA = 0.05). In the measurement model, the latent variables of mothers’ stress during COVID-19 and mindful parenting were significantly associated with the manifest variables involving the respective subscales (*p*s < 0.001), respectively. As for the structural model, the exogenous variable of mindful parenting was negatively related to mothers’ stress during COVID-19 (*β* =  − 0.27, *p* < 0.01). Mothers’ stress during COVID-19 was not related to children’s prosocial behavior (*β* = 0.17, *p* = 0.051), externalizing problems (*β* =  − 0.02, *p* = 0.82), and internalizing problems (*β* = 0.06, *p* = 0.45), after controlling for children’s baseline prosocial behavior, externalizing problems, and internalizing problems (*p*s < 0.001). Children’s sex, children’s age, and household income were entered as covariates of the variables under study. Hence, mothers’ stress during COVID-19 did not mediate between mindful parenting and child adjustment.

### Mindful Parenting as a Moderator

**Fig. 4:**
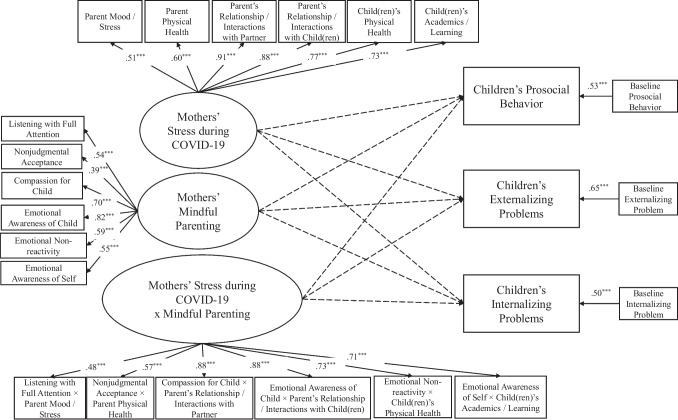
Final model of mindful parenting as a moderator between mothers’ stress during COVID-19 and child adjustment outcomes. This model reflects the results based on the original measure of children’s prosocial behavior. *χ*^2^(277) = 465.34, *p* < 0.001, CFI = 0.96, TLI = 0.95, RMSEA = 0.07. Household income, children’s age, children’s sex were included as covariates for child adjustment but are not depicted in the figure for clarity. Non-significant paths are depicted dashed arrows for clarity. ^*^p < .05, ^**^p < .01, ^***^p < .001

The moderation model fit adequately to the data (*χ*^2^(277) = 465.34, *p* < 0.001, CFI = 0.96, TLI = 0.95, RMSEA = 0.07). Specifically, the latent variables of mothers’ stress during COVID-19 and mindful parenting were significantly associated with the manifest variables involving the respective subscales (*p*s < 0.001), respectively. The latent interaction variable between mothers’ stress during COVID-19 and mindful parenting was also significantly associated with the manifest interaction variables. However, after controlling for the effects of children’s sex, children’s age, household income, and baseline measures of child adjustment, neither the main effects nor the interaction effect of mothers’ stress during COVID-19 and mindful parenting on children’s prosocial behavior, externalizing problems, and internalizing problems was significant (*p*s > 0.05) (see Fig. 4 for details). In the supplementary analyses, the model involving two excluded items of prosocial behavior indicated a similar model fit to the data (*χ*^2^(277) = 457.46, *p* < 0.001, CFI = 0.96, TLI = 0.95, RMSEA = 0.07). However, neither the main effects nor the interaction effect of mothers’ stress during COVID-19 and mindful parenting on children’s prosocial behavior, externalizing problems, and internalizing problems was significant (*p*s > 0.05).

To ensure that the null finding was not due to the method of analysis (i.e., manually creating the observed interaction variables), a second method was used to verify the findings by adding a latent interaction term between mothers’ stress during COVID-19 and mindful parenting within the MPLUS environment (Muthén & Muthén, 1998–2017). The findings converged to indicate that the interaction effects of mothers’ stress during COVID-19 and mindful parenting on children’s prosocial behavior (*B* = 0.01, *SE* = 0.42, *p* = 0.99), externalizing problems (*B* =  − 0.18, *SE* = 0.20, *p* = 0.36), and internalizing problems (*B* =  − 0.15, *SE* = 0.18, *p* = 0.41) were not significant. In the supplementary analyses, the model involving two excluded items of prosocial behavior indicated a similar model fit. The interaction effects of mothers’ stress during COVID-19 and mindful parenting on children’s prosocial behavior (*B* = 0.01, *SE* = 0.38, *p* = 0.98), externalizing problems (*B* =  − 0.18, *SE* = 0.19, *p* = 0.33), and internalizing problems (*B* =  − 0.15, *SE* = 0.18, *p* = 0.39) were not significant.

## Discussion

Grounded in theories of family risks (e.g., Prime et al., 2020; Repetti et al., 2002) and drawing from previous research (e.g., Kelly et al., 2022; C. Wang et al., 2022), this study investigated the associations between mothers’ stress during COVID-19, mindful parenting, and child adjustment. Our findings supported the mediation model, in that mothers’ mindful parenting practices mediated between their perceived stress during COVID-19 and child adjustment, including prosocial behavior, externalizing problems, and internalizing problems (see Fig. 3). As such, mindful parenting was potentially a mechanism that explained why mothers’ stress was linked to child adjustment during the pandemic. On the contrary, mindful parenting did not moderate the relation between mothers’ stress during COVID-19 and child adjustment (see Fig. 4). That is, the link between mothers’ stress during COVID-19 and child adjustment was not dependent on the level of mindful parenting.

Consistent with previous studies showing the link between mothers’ stress and mindful parenting (Cheung et al., 2019; Fernandes et al., 2021; Moreira et al., 2019), the present study indicated that mothers’ greater stress during COVID-19 was associated with fewer mindful parenting practices, as indexed by their lower ability to listen to their child and themselves with full intention, to accept nonjudgmentally the parenting experiences, to be aware emotionally of their child and themselves, to develop compassion for the child and themselves, and to have reduced emotional non-reactivity in parenting (Duncan et al., 2009). In the face of uncertainties brought by COVID-19, mothers might have been preoccupied with self-directed and family-directed concerns, from panic buying as a result of the shortage of groceries and sanitizing items (Taylor et al., 2020) to managing disrupted daily routines (Liu et al., 2021) and unemployment (Blustein et al., 2020; Prime et al., 2020). The present findings revealed that mothers’ stress revolving around COVID-19 was linked to their lower capability to be mindful in parenting. They also substantiated previous research conducted in Eastern and Western contexts (e.g., Bögels & Restifo, 2013; Cheung et al., 2021), in that mothers’ lower level of mindful parenting was associated with child maladjustment, as indicated by children’s greater levels of internalizing and externalizing problems, as well as a lower level of prosocial behavior.

Somewhat surprisingly, the moderation hypothesis was not supported by the present findings. In addition, mothers’ stress during COVID-19 and mindful parenting practices did not additively nor interactively predict child adjustment outcomes in the moderation analyses. The null findings were unexpected, particularly between mindful parenting and child adjustment, as they contrasted with the significant simple correlations as shown in Table 1, as well as the significant mindful parenting-child adjustment link in the mediation model. Simply put, the significant contributions of mindful parenting did not bear out when other predictors were included in the moderation analysis. Given the significant correlations between some indicators of mothers’ stress during COVID-19 and mindful parenting (see Table 1), the null findings might have been due, in part, to multicollinearity. In contrast to past research (Cohodes et al., 2021; Giannotti et al., 2022; Spinelli et al., 2020, 2021; Sun et al., 2022), our findings also revealed a lack of direct association between mothers’ stress during COVID-19 and child adjustment across the zero-order correlations and structural equation models. In other words, the present findings not only falsified the moderation hypothesis, but also pointed to inconsistencies with other studies indicating the direct mothers’ stress-child adjustment link (e.g., Cohodes et al., 2021). Hence, future studies with a larger sample and a longitudinal design with multiple time points are necessary to replicate the present findings.

### Limitations and Future Directions

The present findings should be interpreted in light of the limitations. First of all, this study included mother-report of stress, mindful parenting, and child adjustment, thereby leading to method bias (Podsakoff et al., 2012). As remedies, future research could recruit multiple reporters and collect observational and biophysiological data of stress, parenting, and child adjustment. Although sensitivity analysis did not support an alternative mediation model with mothers’ stress during COVID-19 as a mediator, longitudinal studies are necessary to reduce biases and draw conclusions on the directionality of effects (Maxwell & Cole, 2007). Second, Cronbach’s *α* and McDonald’s *ω* were lower than 0.70 for two subscales of IMP (i.e., emotional awareness of child and nonjudgmental acceptance of parental functioning; Duncan et al., 2009) and two subscales of SDQ (i.e., prosocial behavior and internalizing problems; Goodman, 1997). The low internal consistency and reliability coefficient of the IMP emotional awareness of child subscale might have been due, in part, to the fact that the subscale only had 3 items, whereas the IMP nonjudgmental acceptance of parental functioning subscale only had reverse worded items. As for SDQ, previous studies had shown similarly low internal consistency for SDQ subscales in the Chinese context (e.g., Cheung et al., 2021). The low internal inconsistency and reliability implied that the scales might not have reliably measured the variables of interest. Therefore, the present findings should still be interpreted with caution. Third, given that children’s physical interactions with peers were restricted due to school closure and social distancing (e.g., Fegert et al., 2020; The Government of Hong Kong special Administrative Region Press Releases, 2020), some of the items of prosocial behavior subscale of SDQ (Goodman, 1997) might not be applicable to the present findings. Although the findings involving the original vs. the shortened prosocial behavior subscale were similar, further studies should examine children’s prosocial behavior in diverse contexts, such as remote interactions with peers (e.g., remote play and online chat with peers; Luo et al., 2022) and physical interactions between siblings and other family members at home. Fourth, in this study we did not measure other types of stress, such as financial stress and mothers’ pre-existing parenting stress, as control variables. Future studies should control for well-established correlates of stress, mindful parenting, and child adjustment to determine whether mothers’ stress during COVID-19 predicts the criterion variables, over and above other important factors. Fifth, our participants were primarily from major cities and provinces of China who had above-average household income, limiting the generalizability of the findings to the rest of China. Representative and diverse samples from various provinces and cities within China should be included in the future research.

Notwithstanding the above limitations, this study lends support to the mediation effect of mindful parenting between mothers’ stress during COVID-19 and child adjustment. The findings also refuted the hypothesis of mindful parenting as a moderator. Although the present study involved families from China, the findings may also be relevant to families throughout the world. Hence, cross-cultural and longitudinal studies gearing towards mindful parenting during stressful circumstances merit future investigation.

## Data Availability

The dataset analyzed in this article is not publicly available. Requests to access the dataset should be directed to rebecca.cheung@reading.ac.uk.
